# Treatment of diabetic vasculopathy with rosiglitazone and ramipril: Hype or hope?

**DOI:** 10.4103/0973-3930.54287

**Published:** 2009

**Authors:** Sayeeda Rahman, Aziz Al-Shafi Ismail, Abdul Rashid A. Rahman

**Affiliations:** Department of Clinical Sciences, School of Life Sciences, University of Bradford, Bradford, UK; 1 Department of Community Medicine; Universiti Sains Malaysia,16150 Kubang Kerian, Kelantan, Malaysia; 2 Cyberjaya University College of Medical Sciences 63000 Cyberjaya, Malaysia

**Keywords:** Diabetic vasculopathy, ramipril, rosiglitazone

## Abstract

Cardiovascular diseases are responsible for increased morbidity and mortality in people with diabetes. Diabetic macrovasculopathy is associated with structural and functional changes in large arteries, which causes endothelial dysfunction, increased arterial stiffness, or decreased arterial distensability. Diabetic complications can be controlled and avoided by strict glycemic control, maintaining normal lipid profiles, regular physical exercise, adopting a healthy lifestyle and pharmacological interventions. Treatment goals for patients with type 2 diabetes specify targets for glycemia and other cardiometabolic risk factors, for example, hypertension and dyslipidemia. In recent years, special attention has been devoted to both thiazolidindiones (TZDs) and angiotensin converting enzyme (ACE) inhibitors as clinical trials revealed that these drugs may reduce the rate of progression to diabetes or delay the onset of diabetes, regression of impaired glucose tolerance (IGT) to normoglycemia and reduces the composite of all-cause mortality, nonfatal myocardial infarction and stroke in patients with diabetes. This review focuses on the potential roles of rosiglitazone, a member of TZD class of antidiabetic agents, and ramipril, an ACE inhibitor, in preventing the preclinical macrovasculopathy in diabetes and IGT population.

## Introduction

Diabetes is one of the most challenging health problems in the twenty-first century. It is ranked as the fifth leading cause of death and is a major risk factor for various cardiovascular diseases (CVD).[1] Cardiovascular diseases are responsible for more than 50% and up to 80% of deaths in people with diabetes as well as for very substantial morbidity and loss of quality of life[2] [Table 1]. The most important forms of CVD are coronary heart disease, cerebrovascular disease, and peripheral vascular disease. These lead to heart attacks, angina, heart failure, stroke, and gangrene or ulceration of the feet and legs requiring amputation. People with diabetes are also prone to developing CVD at a younger age and having more severe effects than people without diabetes. In addition, risk is increased even at the earlier stages of glucose intolerance.

**Table 1:** Cardiovascular diseases and diabetes: Double jeopardy[2]

Approximately 80% of people with diabetes die of CVD. On average, people with type 2 diabetes will die 5-10 years before people without diabetes and most of this excess mortality is due to CVD. People with type 2 diabetes are over twice as likely to have a heart attack or stroke as people who do not have diabetes. Indeed, people with type 2 diabetes are as likely to suffer a heart attack as people without diabetes who have already had a heart attack. Strokes occur twice as often in people with diabetes and hypertension as in those with hypertension alone. People with diabetes are 15-40 times more likely to have a lower limb amputation compared to the general population. People with diabetes have two to four times the risk of developing atherosclerosis compared to people without diabetes. The treatment of CVD accounts for a large part of the huge healthcare costs attributable to type 2 diabetes, that have been estimated to account for 10-12% of European health care expenditure. Part of the CV risk associated with IGT and diabetes is undoubtedly due to their association with other CV factors such as hypertension, high LDL-cholesterol and low HDL-cholesterol, and smoking. Lifestyle changes that improve blood glucose control, for example weight loss, dietary changes, and increased physical activity are also likely to improve these other CV risk factors.

### Diabetic vasculopathy

**Figure 1:**
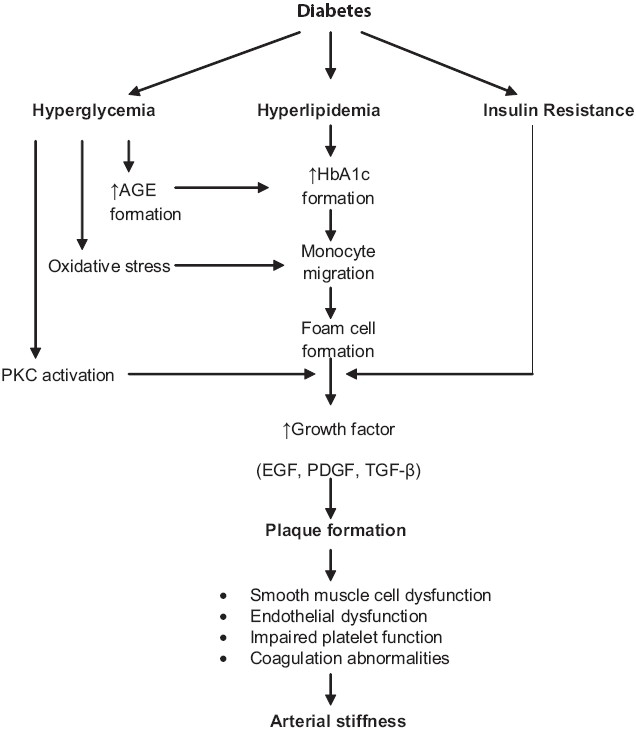
Pathogenesis and pathophysiology of diabetic macrovasculopathy

Diabetes mellitus is a multifactorial disease associated with a number of microvascular (retinopathy, neuropathy, and nephropathy) and macrovascular complications.[3,4] Diabetic macrovasculopathy is associated with structural and functional changes in large arteries that lead to increased stiffness, abnormal pulse wave travel, and systolic hypertension.[4] Structural changes mainly result from glycation of wall components and functional changes originate in endothelial dysfunction, increased arterial stiffness or decreased arterial distensibility [Figure 1]. These changes promote the development of left ventricular hypertrophy, an independent risk factor for cardiovascular (CV) mortality.[5] Apart from the above-mentioned mechanisms, metabolic [advanced glycation end production (AGE), cytokines], humoral (renin-angiotensin system, endothelin, sympathetic nervous system) and hemodynamic (arterial hypertension and mechanical strain) factors contribute to the characteristic dysfunction in diabetic vasculopathy.[6] The initiators of vasculopathy that ultimately develop into long-term diabetic complications can be controlled and avoided by strict glycemic control, maintaining normal lipid profiles, regular physical exercise, adopting a healthy lifestyle and pharmacological interventions.

### Treatment Modalities of Type 2 Diabetes

As the prevalence of type 2 diabetes continues to increase worldwide, there is an enhanced need for effective disease management. The International Diabetes Federation (IDF) has recently introduced new global guidelines for the management of diabetes.[7] Three modalities of treatment are currently available to manage diabetes: lifestyle modification including appropriate diet and exercise programs, oral anti-diabetic agents, and insulin. Patients with diabetes are insulin resistant and often have metabolic syndrome, which requires a multifactorial intervention in order to reduce the incidence of CV complications[8] [Table 2]. Treatment goals for patients with type 2 diabetes specify targets for glycemia and other cardiometabolic risk factors, for example, hypertension and dyslipidemia[7,9] [Table 3].

**Table 2:** Control of cardiometabolic parameters in the management of type 2 diabetes as recommended by IDF[8]

Cardiometabolic parameters	Target values
Glycemia	
Prebreakfast and premain	
evening-meal glucose	< 6.0 mmol/l (<110 mg/dl)
BP	<130/80 mmHg
Lipids	
LDL-C	< 2.5 mmol/l (95 mg/dl)
HDL-C	> 1.0 mmol/l (_40 mg/dl)
Triglycerides	< 2.3 mmol/l (<200 mg/dl)

**Table 3:** Treatment modalities of type 2 diabetes

Cardio-metabolic abnormalities	Drugs	Mode of action
Hyperglycemia Insulin resistance	Biguanides	Increases liver and muscle insulin sensitivity; decreases hepatic glucose production
	Sulphonylureas	Insulin secretogogues
	Alpha-glucosidase inhibitors	Delay the absorption of polysaccharides and also act to attenuate postprandial glucose excursions
	Sulphonylurea-like agents	Insulin secretogogues
	Thiazolidinediones	Insulin sensitizers that improve glucose uptake in adipose tissues and skeletal muscles
	Insulin	Reduces hepatic glucose output and increases peripheral glucose utilization
Hypertension	ACE inhibitors	Block the formation of AT-II, increase bradykinin level. As a result reduce vasoconstriction, reduce sodium and water retension, and increase vasodilation (through bradykinin).
	Angiotensin receptor blockers Losartan and valsartan	Competitive inhibition of AT-II receptor (Type 1). Effect more specific on AT-II action, less or none on bradykinin production or metabolism.
	Beta blockers	Inhibit renin release and AT-II and aldosterone production and lower peripheral resistance; may decrease adrenergic outflow from the CNS.
	Calcium channel blockers	Dilate peripheral arterioles and thereby reduce BP by inhibiting calcium influx into arterial SM cells.
	Diuretics	Lower BP by depleting body sodium stores resulting in reduction of total blood volume and cardiac output; initially peripheral vascular resistance increases but declines when CO returns to normal level (6-8 weeks)
Dyslipidemia	Statins	Increase lipid profile and decrease atherogenic tendency. Lower LDL-C, improve TC:HDL-C, lower apo B.
	Fibric acid derivatives	Increase lipid profile and decrease atherogenic tendency. Lower TGs, raise HDL-C, lower TC:HDL-C and shift LDL from smaller to larger particles.
Platelet activation and	Aspirin	Antiplatelet effect
aggregation	Clopidogrel	Irreversible blockade of the adenosine diphosphate (ADP) receptor on platelet cell membranes
	Ticlopidine	Interferes with platelet membrane function

In recent years, special attention has been devoted to both thiazolidinediones (TZDs) and angiotensin converting enzyme (ACE) inhibitors when TRIPOD study[10] demonstrated that troglitazone may reduce the rate of progression to diabetes in women diagnosed with gestational diabetes and HOPE Study[11] showed that ramipril may delay the onset of diabetes. The landmark study ProActive (PROspective pioglitAzone Clinical Trial In macroVascular Events) demonstrated that pioglitazone reduces the composite of all-cause mortality, nonfatal myocardial infarction, and stroke in patients with T2DM who have a high risk of macrovascular events.[12] Recently, published landmark DREAM study demonstrated that rosiglitazone has a substantial benefit on prevention of diabetes and regression to normoglycemia and ramipril has a modest benefit on regression to normoglycemia.[13,14]

The TZDs are new oral antidiabetic agents providing a novel means to reduce hyperglycemia by improving insulin sensitivity. Moreover, TZDs have vasculoprotective properties beyond glycemic control.[15] These drugs have potentially favorable effects on other components of the insulin resistance syndrome. As insulin sensitizers, they may modify CV risk factors and reduce CV mortality in T2DM and insulin resistance subjects.[16] The ACE inhibitors therapy reduces both microvascular and macrovascular complications in diabetes and appears to improve insulin sensitivity and glucose metabolism.[17] This review focuses on the potential roles of rosiglitazone, a member of TZD class of antidiabetic agents, and ramipril, an ACE inhibitor, in preventing the preclinical macrovasculopathy in diabetes and IGT population.

Rosiglitazone is a highly selective and potent agonist for the peroxisome proliferator-activated receptor-gamma (PPARγ). These proliferator-activated receptors are found in key target tissues for insulin action such as adipose tissue, skeletal muscle, and liver. Activation of PPARγ nuclear receptors regulates the transcription of insulin responsive genes involved in the control of glucose production, transport, and utilization. In addition, PPARγ-responsive genes also participate in the regulation of fatty acid metabolism. Ramipril has direct effects on the renin-angiotensin-kallikrein system and may prevent diabetes through effects on the beta cell and by vascular and metabolic effects on muscle that may amplify the effects of insulin.[18] The ACE inhibitors increase islet blood flow and pancreatic beta-cell perfusion by reducing angiotensin II-mediated vasoconstriction in the pancreas,[19] which may potentially slow down or reverse the decline in beta-cell function. The ACE inhibitors may also increase the insulin-mediated glucose disposal, thereby decreasing the need for pancreatic insulin secretion and may reduce insulin resistance in skeletal muscle.[20] This may be due to increased bradykinin-mediated nitric oxide production.[21] The ACE inhibitors may also reduce insulin resistance at the liver and fat cell, which reduce hepatic glucose production and lower free fatty acid level.[22]

### Randomized Controlled Trials with Rosiglitazone and Ramipril: Effects on Diabetes

A number of clinical trials were conducted to investigate the efficacy of rosiglitazone in improving the glycemic status in type 2 diabetes and IGT patients [Table 4]. As monotherapy, three 8-12 weeks dose-finding placebo-controlled randomized trials were found to reduce fasting plasma glucose (FPG) and HbA1c levels with rosiglitazone 4-12 mg/day.[23–25] In two 26-week placebo-controlled studies,[26,27] significant reductions in FPG and HbA1c were seen with rosiglitazone. A 52-week randomized, double-blind trials showed that FPG and HbA1c levels fell significantly with rosiglitazone in comparison to glibenclamide.[28] A recently published study demonstrated that rosiglitazone therapy reduced plasma insulin, proinsulin, split proinsulin, and free fatty acid level compared with glibenclamide therapy.[29]

**Table 4:** Monotherapy clinical trials on rosiglitazone in T2DM patients

Researchers	Study population	Methodology	Results/comments
Nolan *et al.*[23]	T2DM; *n*=380	Rosiglitazone 4, 8, or 12 mg q.i.d.; duration: 8 weeks	All doses lowered FPG significantly
Patel *et al.*[24]	T2DM; *n*=380	Rosiglitazone 0.05, 0.25, 1, 2 mg twice daily; duration: 12 weeks	FPG was reduced significantly by rosiglitazone 1 and 2 mg b.i.d. Only 2 mg b.i.d. produced a significant reduction HbA1c
Raskin *et al.*[25]	T2DM; *n*=30	Rosiglitazone 2, 4, 6 mg b.i.d.; duration: 38 weeks	Significantly reduced FPG and postprandial glucose, C-peptide and insulin with rosiglitazone 4 mg b.i.d.
Phillips *et al.*[26]	T2DM; *n*= 959	Rosiglitazone 4 mg o.d.,2 mg b.i.d, 4 mg b.i.d.,8 mg o.d.; duration: 26 weeks	Produced drug-dependent reduction in HbA1c
Lebovitz *et al.*[27]	T2DM; *n*= 493	Rosiglitazone 2 or 4 mg b.i.d.; duration: 26 weeks	Rosiglitazone 2 and 4 mg b.i.d decreased mean HbA1cand FPG
Charbonnel *et al.*[28]	T2DM; *n*=587	Rosiglitazone 2, 4 mg b.i.d and glibenclamide (15 mg/day); duration: 52 weeks	At week 52, significant decrease in mean HbA1cand FPG
Hanefeld *et al.*[29]	T2DM; *n*=598	Rosiglitazone 4, 8mg/day or glibenclamide 15mg/day; duration: 52 weeks	Rosiglitazone therapy reduced plasma insulin, proinsulin, split proinsulin and free fatty acid level compared with glibenclamide

A recent publication of a meta-analysis of data from 42 clinical trials suggesting an increased risk of myocardial infarction (MI) and cardiovascular death with rosiglitazone led to a media furor and widespread patient panic.[30] The pooled data showed a 43% increase in relative risk of MI among T2DM treated with rosiglitazone. However, interim findings from ongoing RECORD (rosiglitazone evaluated for cardiac outcomes and regulation of glycemia in diabetes) study were inconclusive regarding the effect of rosiglitazone on the overall risk of hospitalization or death from cardiovascular causes.[31] The study found no evidence of any increased mortality, either from any cause or from cardiovascular causes. Further research is needed to determine the long-term cardiovascular effects of rosiglitazone.

Diabetes-preventive benefits have also been claimed by a number of previous studies with angiotensin-converting enzyme (ACE) inhibitors. The HOPE study was the first to explore that ACE inhibitor ramipril prevents the development of diabetes.[11] The possibility that reduce the number of new cases of diabetes was also supported by a number of other studies.[32–36] As monotherapy, two trials have evaluated the use of ramipril in diabetic patients [Table 5]. Trevisan and Tiengo[37] showed that low dose ACE inhibition with ramipril could arrest the progressive rise in albuminuria in diabetic patients with persistent microalbuminuria. The beneficial effects of this therapy were accompanied by relatively few adverse events and none of them was directly related to treatment. Another study conducted by Nielsen *et al*,[38] demonstrated that ramipril induces regression of left ventricular hypertrophy in normotensive, nonalbuminuric NIDDM patients, independent of reduction in systemic blood pressure.

**Table 5:** Monotherapy clinical trials on Ramipril in T2DM patients

Researchers	Study population	Methodology	Results/Comment
Trevisan and Tiengo[37]	T2DM; n=122	Ramipril 1.25 mg/day; duration: 6 months	Low-dose ACE inhibition with ramipril could arrest the progressive rise in albuminuria in T2DM patients with persistent microalbuminuria.
Nielsen *et al.*[38]	T2DM, n= 16	Ramipril (5mg)/day; duration: 6 month	Beneficial impact of ramipril on left ventricular hypertrophy in normotensive nonalbuminuric T2DM patients
MICROHOPE Substudy[39]	T2DM; n=3577	Ramipril 10 mg/day vs. placebo and Vitamin E; duration: 4.5 years	Lowered the risk of the combined primary outcome by 25%, myocardial infarction by 22%, stroke by 33%, CV death by 37%, total mortality by 24%, revascularization by 17%, and overt nephropathy by 24%.
			Ramipril was beneficial for CV events and overt nephropathy in T2DM patients

T2DM= Type 2 Diabetes Mellitus

### Rosiglitazone and Preclinical Vasculopathy

A number of clinical studies involving patients with T2DM demonstrated the antiatherogenic effect of TZDs involving troglitazone,[40–42] pioglitazone[43–46] and rosiglitazone.[47–49] The clinical studies on vasculopathy with rosiglitazone are summarized in Table 6.

**Table 6:** Studies investigating the effect of rosiglitazone and ramipril on arterial stiffness

Researchers	Study population	Methodology	Comments
Kim *et al.*[47]	Prediabetes (n=50) or nondiabetic metabolic syndrome (n=49)	Rosiglitazone 4 mg/day; Duration: 12 weeks. brachial-ankle PWV and adiponectin levels; volume plethymographic apparatus	PWV was significantly decreased in the rosiglitazone group in comparison to baseline
Shargorodsky *et al.*[48]	T2DM; n= 52	Rosiglitazone of 4 mg/day; duration: 6 months; large and small artery elasticity; pulse wave contour analysis	Significant change was observed in small artery elasticity but no difference in large artery elasticity
Pistroch *et al.*[49]	T2DM; n=12	Rosiglitazone (4 mg b.i.d) with nateglinide; duration: 12 weeks; endothelial dysfunction; venous occlusion plethysmography	Rosiglitazone had therapeutic effects on endothelial dysfunction in T2DM patients
Lonn *et al.*[53]	T2DM; n=732	Ramipril 2.5 mg/d or 10 mg/d and vitamin E or their matching placebo; duration: 4.5 years; intima-media thickness (IMT); B-mode carotid ultrasound	Ramipril 10 mg significantly reduced progression of carotid artery wall thickness
Rahman *et al.*[51]	Newly diagnosed, never treated T2DM (n=33) and IGT (n=33)	Rosiglitazone 4mg/day or Ramipril 5 mg/d or placebo; duration: 1 year; PWV and AI; Sphygmocor	Rosiglitazone significantly decreased PWV and AI and ramipril significantly reduced AI in IGT patients.

A study by Kim *et al*.[47] evaluated the effect of rosiglitazone in subjects with prediabetes or nondiabetic metabolic syndrome and demonstrated significant decrease in arterial stiffness (PWV) in the rosiglitazone group in comparison to untreated control group. The observed PWV change might have resulted from additional effects of rosiglitazone beyond metabolic control. Other studies also showed that rosiglitazone in healthy subjects[50] and in T2DM patients[49] significantly improved vascular endothelial function without changes in blood glucose level. A possible explanation of reduced arterial stiffness might be that rosiglitazone directly affects PPAR-γ activation in the vascular wall.[47] Another study by Shargorodsky *et al*.[48] demonstrated significant improvement of the small artery elasticity with rosiglitazone; however, no significant change was found in the large artery elasticity. The authors explained that large arteries have a major component of fixed fibrotic tissue that probably needs more time for repair. Pistroch *et al*.[49] compared the glycemic control by rosiglitazone with nateglinide and demonstrated that rosiglitazone had therapeutic effects on endothelial dysfunction in diabetic patients.

Until now, no research has been published to examine the effect of rosiglitazone on arterial stiffness in IGT patients. The DREAM trial examined the effect of rosiglitazone on atherosclerosis on IGT, measured by sequential carotid ultrasound in a subset of DREAM participants, which is yet to be published.[13] A recent study by Rahman *et al*.[51] showed that rosiglitazone significantly reversed preclinical vasculopathy in newly diagnosed, never treated IGT individuals as evident by significant decrease in PWV and AI after 1 year of treatment.

### Ramipril and Preclinical Vasculopathy

Studies that have focused on the effects of antiatherogenic effect of ACE inhibitors on diabetes are scarce and no research was done to examine the effect of ramipril on arterial stiffness in IGT patients. The antiatherogenic properties of ACE inhibitors may be mediated by the lowering of angiotensin-II and the increasing of bradykinin concentrations. These result in decreased proliferation and migration of smooth muscle cells, decreased accumulation and activation of inflammatory cells, decreased oxidative stress, and increased endothelial nitric oxide formation, leading to improved endothelial function. The observed benefits of ramipril may be largely due to a protective effect of ACE inhibitors on the arterial wall.[52] The clinical studies on vasculopathy with ramipril are summarized in Table 6.

The MICRO-HOPE trial, a substudy of the HOPE trial, is first to examine the cardioprotective effects of ramipril on diabetic patients.[39] The study reported that ramipril was beneficial for CV events and overt nephropathy in people with diabetes. The CV benefit was greater than that attributable to the decrease in blood pressure. The SECURE trial,[53] a substudy of the HOPE Study, has examined the effect of ramipril on intima-media thickening. The study found that treatment with ramipril significantly reduced the progression of carotid artery wall thickness. In a recent study, Rahman *et al*.[51] found that ramipril reduced large artery stiffness as shown by significant decrease of AI after 1 year of treatment in newly diagnosed, never treated IGT individuals.

## Conclusion

Vascular complications are the major causes of morbidity and mortality in patients with diabetes. Limited numbers of studies with diabetic patients have shown the beneficial effects of rosiglitazone and ramipril on diabetic vasculopathy. Research finding established the fact that both drugs have the potentiality to offer novel therapeutic strategies to prediabetic vasculopathy in diabetes and IGT patients because of their antiatherogenic effects. Clinical trials are needed with IGT patients as more than 8% of adult populations worldwide have either IGT or IFG[54] and every year about 5-10% of these people would develop diabetes who would be at high risk for several chronic complications. It is noteworthy that even at the stage of IGT, before full-blown diabetes has developed, the risk of CVD is already increased by about two times compared to people with normal glucose tolerance.[55] Unless diabetic macrovasculopathy in patients with IGT are identified and treated, the enhanced risk of macrovascular complications will increase in future.[56] Further randomized controlled trials should be undertaken to show whether rosiglitazone and ramipril can prevent/reverse the preclinical vasculopathy both in diabetic and in IGT patients.
